# The study to identify the psychological factors that influence anxiety in mine workers

**DOI:** 10.1192/j.eurpsy.2025.1034

**Published:** 2025-08-26

**Authors:** P. Sarantuya, B. Purev, M. Dashdondov, B. Dashdorj, A. Altantsetseg

**Affiliations:** 1 Medical department; 2 Basic Medical Department, Etugen University, Ulaanbaatar, Mongolia

## Abstract

**Introduction:**

If stress, insomnia, anxiety, and depression persist for a long time and occur more than normal, it can become a serious illness. Based on the data from 263 mountain work reports submitted to the Department of Mining, Production, and Technology and the Coal Research Department of the Mineral Oil Department in 2018, a human resource survey of the industry was conducted. There are 56,635 registered workers in the mining sector, 96% of whom are Mongolian and 4% are foreign experts. 90% of all employees working in the mining industry are men, and 10% are women. In our country, there is a lack of research on the psychological health of mine workers and the factors affecting it, so this study is the reason for conducting this research.

**Objectives:**

To identify the anxiety of mine workers and to study some correlations and effects.

**Methods:**

The survey was conducted from March 26, 2024, to April 5, 2024, by the employees of “TiTiGiViSiOu LLC,” located in Tsogtsetsii Sum, Umnugobi Province, according to GAD7, SRQ20, issued by WHO for doctors of primary health care institutions. Survey data were collected from mining workers using the PHQ9, 18 sleep disturbance questionnaires, and 10 general information questionnaires. IBM SPSS-26 software was used to analyze the research.

**Results:**

66.0% of the study participants had no anxiety, 23.3% had mild anxiety, 6.8% had moderate anxiety, and 3.9% had severe anxiety. Also, 70.9% work in the night shift, while 24.3% do not work in the night shift. However, 4.8% answered that they sometimes work the night shift. The majority of night shift workers are male workers. 66.0% of the respondents had no anxiety, 23.3% had mild anxiety, 6.8% had moderate anxiety, and 3.9% had severe anxiety. Anxiety has a strong direct correlation with stress (r = 0.7**, p = 0.00), depression (r = 0.87**, p = 0.00), and sleep disturbance (r = 0.58**, p = 0.00). degree was directly related. In univariate regression analysis, a one-point increase in stress score was associated with a 71% increase in anxiety. The depression score increased by 80% per unit increase, and the sleep disturbance score increased by 87% per unit increase. Multivariate linear regression analysis revealed that anxiety increased by 18.6% when the stress score increased by one and depression increased by 66.2% when the depression score increased by one.

**Conclusions:**

Anxiety is strongly associated with stress, depression and sleep disturbances. An increase in stress, depression, and sleep disturbance scores leads to an increase in anxiety. Multivariate linear regression analysis reveals these relationships.

**Disclosure of Interest:**

None Declared

